# Creutzfeldt-Jakob Disease in South Texas: A Case Series of Three Hispanic Patients

**DOI:** 10.7759/cureus.85699

**Published:** 2025-06-10

**Authors:** Daniela I Salinas, Roberto A Cruz, Silvia Rincon Rueda, Leonel J Estofan

**Affiliations:** 1 Neurology Institute, Doctors Hospital at Renaissance, McAllen, USA; 2 Neurology, Tecnologico de Monterrey, Monterrey, MEX; 3 Neurology, Universidad Industrial de Santander, Bucaramanga, COL

**Keywords:** creutzfeldt-jakob disease, neuro-immunology, prion diseases, rapidly progressive dementia, rare neurodegenerative disease

## Abstract

Creutzfeldt-Jakob disease (CJD) is a rare, rapidly progressive neurodegenerative disorder instigated by the pleating of prion proteins. Most cases are sporadic, with no identifiable genetic or environmental trigger.

This retrospective case series aims to bridge the current knowledge gap in regard to CJD in Hispanic populations. We hope to review the management and outcomes of three Hispanic patients diagnosed with sporadic CJD at a community hospital in South Texas between 2021 and 2024. Clinical data, imaging studies, and cerebrospinal fluid (CSF) results were reviewed for three unrelated patients aged over 55 who met the Centers for Disease Control and Prevention (CDC) criteria for probable CJD.

All patients presented with progressive cognitive decline, cerebellar signs, and myoclonus. Magnetic resonance imaging (MRI) showed cortical ribboning; CSF was positive for the 14-3-3 protein and/or real-time quaking-induced conversion (RT-QuIC). All patients died within two years of symptom onset.

These cases highlight the need for specialized medical personnel for the early recognition of CJD symptoms in underrepresented populations. Improved awareness and diagnostic readiness can enhance care planning and avoid unnecessary delays from symptom onset to diagnosis and care.

## Introduction

Prion diseases are rare, fatal neurodegenerative disorders that affect the central nervous system (CNS) due to the accumulation of misfolded proteins (PrP) within the neurons. Creutzfeldt-Jakob disease (CJD) is the most common prion disease and typically results in death within one year. The misfolded prion proteins convert normal proteins into this pathogenic form, leading to rapid neurologic decline [1,2].

CJD has four subclassifications: sporadic (sCJD), genetic (gCJD), iatrogenic (iCJD), and variant (vCJD), with sCJD accounting for approximately 85-90% of cases [3]. CJD has a global incidence of approximately one case per million per year [4]. Individuals aged 55 and older are most commonly affected, with no apparent preference for gender or ethnicity [4,5]. Early suspicion for diagnosis relies heavily on the ability of healthcare providers to recognize a specific clinical presentation. According to CDC criteria, probable CJD requires at least two of the following: myoclonus, visual or cerebellar signs, pyramidal/extrapyramidal signs, or akinetic mutism; plus a supportive test, such as positive 14-3-3 protein or RT-QuIC, characteristic EEG, or cortical ribboning on MRI [6]. RT-QuIC has improved antemortem diagnostic accuracy, although definitive diagnosis requires neuropathological confirmation.

Despite extensive research, data on CJD in the Hispanic population are difficult to obtain. Reports from Mexico (29 cases between 1990-2020) and Argentina (211 cases between 1997-2008) suggest severe underdiagnosis relative to global estimates [7,8]. This study contributes novel clinical observations from a Hispanic-majority region in the U.S., where awareness and resources for diagnosing rare neurodegenerative diseases may be limited.

Given these challenges, our case series offers valuable clinical insights. The objective of this study is to describe the clinical features, diagnostic workup, and disease progression in three unrelated Hispanic patients diagnosed with probable sCJD at a community hospital in South Texas.

## Case presentation

Three unrelated Hispanic patients aged over 55 were included. All met CDC criteria for probable CJD and had congruent imaging and CSF findings. Patients with alternative diagnoses or who did not meet CDC criteria were excluded. None of the patients reported a travel history to the United Kingdom (UK).

Case 1

A 65-year-old male with a relevant past medical history of hyperlipidemia, type II diabetes mellitus, and hypertension initially approached his primary care physician (PCP) in July 2023 due to persistent dizziness, intermittent headaches, and confusion over two weeks. Four days before his initial appointment, there was an additional onset of gait abnormalities and right arm involuntary movement and weakness. The patient chose to go to an emergency department, where he was treated with pain relief medication to aid his headache.

During the mental status exam, the patient was alert and oriented to person, place, and time. His speech was spontaneous and fluent. The physical exam revealed motor symptoms comprising spastic hypertonia and hyperreflexia throughout the right side. Appendicular ataxia and rhythmic eye movements could be appreciated, making epilepsy a differential diagnosis. There were no sensory alterations during the gross exam.

**Figure 1:**
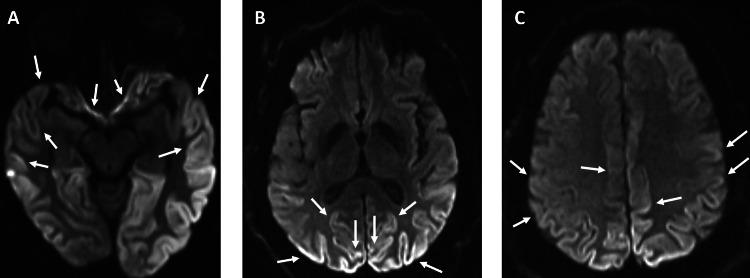
MRI (axial view) of Case 1 with cortical hyperintensity in DWI sequence The arrows point to the cortical ribboning of the bilateral (A) temporal, (B) occipital, and (C) parietal lobes. DWI: diffusion-weighted imaging

The MRI revealed symmetric bilateral parietal and occipital lobe cortical restricted diffusion on diffusion-weighted imaging (DWI) (Figure 1). The patient was started on an antiepileptic, which quickly waned in its efficacy. Benzodiazepines and steroids were trialed; however, symptoms worsened to include ataxic gait and left leg weakness. An electroencephalograph (EEG) was performed, but the findings were nonspecific and did not aid in diagnosis.

With a high suspicion for CJD, a lumbar puncture (LP) with quantifying protein 14-3-3 gamma (γ) for the suspected diagnosis was performed. This test typically requires two weeks for results to be available. Elevated protein values in the CSF sample and T2 hyperintensities in the brain MRI raised suspicion for autoimmune encephalitis. This prompted interventions, including plasmapheresis and intravenous Immunoglobulin (IVIg). The autoimmune encephalitis CSF laboratory panel arrived before the CJD panel, with negative results. The patient’s family noted his decline through worsening weakness of the right upper extremity and the inability to ambulate due to severe dizziness and myoclonus. He was able to carry out non-fluent conversations with a clear understanding. He recalled one of the last three U.S. presidents and was not able to recall his wife’s date of birth. 

The patient experienced waxing and waning lucid intervals and was no longer able to return to a functional baseline. The CSF results for CJD revealed a high likelihood of prion disease with positive real-time quaking-induced conversion (RT-QuIC) results and an elevated 14-3-3γ value at 6855 ng/mL. These results were congruent with the CDC criteria for a probable diagnosis of Creutzfeldt-Jakob disease. 

Case 2

A 58-year-old male with an otherwise unremarkable past medical history presented with three months of short-term memory problems beginning in October 2021. Vertigo soon accompanied his memory loss and was initially described as mild and intermittent, gradually becoming constant. Moreover, the patient's spouse began to notice a decline in his cognitive abilities, which markedly affected his daily home and work life. This patient worked as a surgeon with a high daily volume of patients. He complained of being forced to slow down his practice due to an inability to keep up with his usual performance. The cognitive decline became noticeable when he was unable to complete short surgical procedures in the usual allotted time. 

**Figure 2:**
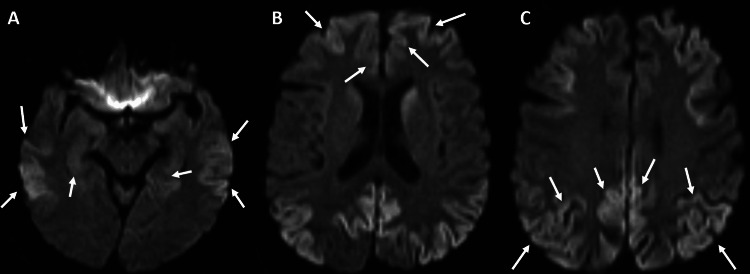
MRI (axial view) of Case 2 with cortical hyperintensity in DWI sequence The arrows point to the cortical ribboning of the bilateral (A) temporal, (B) frontal, and (C) parietal lobes. DWI: diffusion-weighted imaging

He was very physically active but promptly became incapable of completing a workout regimen. Personality changes, such as increased irritability, were worrisome to his spouse. On multiple occasions, he locked himself out of his house and would get lost while driving his usual route. The physical exam of Patient 2 revealed standard bulk and tone of all muscle groups, normal reflexes, absence of tremors, and ataxic gait during this appointment. His ataxic gait manifested with concurrent dizziness, and a brain MRI with/without contrast was deemed necessary by the neurology service, which showed scattered foci of abnormal high T2 signal intensity in fluid-attenuated inversion recovery (FLAIR). Significant diffusion restriction within the cortex was observed (Figure 2). Other tests included long-term EEG recording, lumbar puncture with CSF analysis, an autoimmune encephalitis panel, and a neuron-specific enolase biomarker measure.

**Figure 3:**
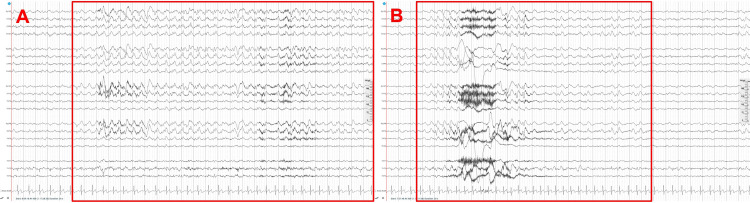
EEG of Patient 2 The EEG readings within the red squares showed the sharp triphasic waves in (A) Fp1-F7 and Fp2-F8. This progressed to epileptiform activity involving (B) all cerebral lobes.

By February 2022, Patient 2 was oriented to self and place but not to time, and was not able to complete mental serial subtractions. There were no language alterations. The cranial nerve exam was relevant for nystagmus on the right-sided gaze. The patient did not present with any new motor, reflex, or cerebellar lesions. The patient's ataxic gait persisted with a positive Romberg sign. Lumbar puncture with CSF analysis showed elevated protein at 69 mg/dL, no unique oligoclonal bands, normal culture findings, normal cytology, and a negative autoimmune encephalitis panel. The paraneoplastic panel was negative for antibodies Anti-Yo, Anti-Ri, Anti-Hu, and Anti-Aquaporin 4. Long-term EEG recordings showed triphasic wave complexes and generalized epileptiform discharges (Figure 3). Neuron-specific enolase (NSE) protein was elevated at 24.3 ng/mL. All of these findings led to the probable diagnosis of Creutzfeldt-Jakob disease.

March 2022 saw the addition of severe dementia, hallucinations, global aphasia, agitation, spasticity, and rigidity. In a matter of days, he became bed-bound. The patient’s immediate family decided that the best course of action would be to transition him to hospice care to provide the best quality of life possible for the patient's remaining time.

Case 3

A 66-year-old female with a past medical history of type II diabetes mellitus and arterial hypertension was referred to our neurology institute in April 2021 for the evaluation of possible encephalitis. Symptoms began in March 2020 when Patient 3 developed slurred speech, word searching, and forgetfulness, which progressed to right-sided weakness within two weeks. Her first MRI was misinterpreted as a multifocal stroke. An ophthalmologic evaluation revealed complete right-eye blindness; subsequent re-evaluation showed left-eye vision deterioration from an unidentified cause. A prominent complaint was a sensation of electrical movement accompanied by sporadic and jerky movements, occasionally leading to falls.

Symptoms waxed and waned, inhibiting the patient's autonomy. She had trouble dressing and moving from her bed to a chair at home. Her speech became slurred, and her gait unsteady with right-arm hypertonia, right-eye ptosis, right-eye blindness, and incontinence. There was a decreased sense of position, vibration, light touch, pinprick, and temperature in both lower extremities from toes to knees. Reflexes were absent on the left side and 1+ on the right knee and brachioradialis. Her gait consisted of small, slow steps with no apparent ataxia. She obtained a score of 12 in her mini-mental exam, consistent with moderate cognitive impairment.

**Figure 4:**
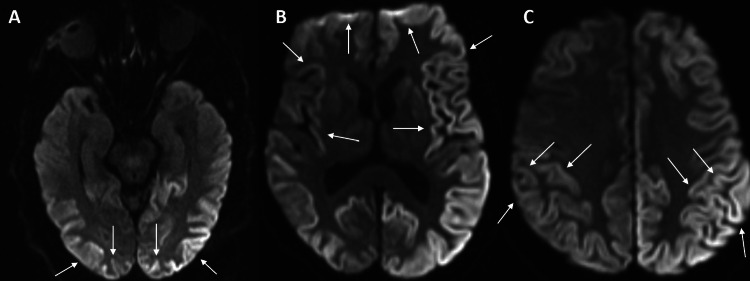
MRI (axial view) of Case 3 with cortical hyperintensity in DWI sequence The arrows point to the cortical ribboning of the bilateral (A) occipital, (B) frontal, temporal, and (C) parietal lobes. DWI: diffusion-weighted imaging

It was concluded that hospitalization would be the best course of action. From this point onward, the patient was no longer able to get up from the bed due to severe spastic weakness. Her latest MRI revealed multiple, scattered T2 hyperintensities and cortical restricted diffusion in the DWI (Figure 4). While waiting for the results of the patient's CSF, a trial of IV methylprednisolone was administered without improvement. In days, the patient developed aphasia, complete blindness, ophthalmoplegia, and rapidly progressive dementia. Her neuron-specific enolase test was elevated at 20.3 ng/mL. The patient's family decided to proceed with home hospice care.

## Discussion

These cases underscore the need for clinical vigilance in communities where prion diseases may not be on the diagnostic radar. Delays in diagnosis were largely due to the initial misinterpretation of nonspecific symptoms, misdiagnosis (e.g., stroke), and logistical delays in specialized CSF testing. Increased training and diagnostic flowcharts for atypical dementias could expedite recognition and reduce patient burden.

From a public health perspective, timely diagnosis facilitates early family counseling and palliative care planning, reducing unnecessary interventions. The occurrence of three CJD cases in one regional hospital over three years may reflect improved detection, referral centralization, or underrecognized local incidence rather than variant forms, which were ruled out by history.

Potential biases include selection bias from a small sample size and referral bias, as all patients were seen at a specialized neurology center. Misclassification was minimized by a thorough differential diagnosis and the consistent use of CDC criteria. However, the absence of postmortem confirmation limits diagnostic certainty.

The three patients were compared in Table 1. The pathophysiology of CJD explains many of its hallmark clinical and EEG findings. The degeneration and disappearance of nerve cells, along with extensive astroglial proliferation, give this disease its characteristic microscopic spongiform appearance. The degeneration of neuronal cells, especially of inhibitory neurons in the thalamic reticular nuclei, could be linked to the occurrence of myoclonus and alterations in the EEG [5]. Once a clinical diagnosis is made, confirmatory studies must be obtained. The 14-3-3 protein has a sensitivity of 92% (95% confidence interval (CI) 89.8-93.6), and a specificity of 80% [9-11]. The prolonged confirmatory diagnosis of CJD plays a detrimental part in overall patient support and their family's opportunity to adapt to the rapid decline of the patient. Patients with CJD require urgent assessment to manage their rapidly increasing needs and end-of-life support [12].

**Table 1:** Diagnostic exams * Positive when a PrP induces the misfolding of normal cellular proteins. PrP: prion protein

		Normal	Case 1	Case 2	Case 3
CSF Findings	Proteins	15-45 mg/dL	104 mg/dL	69 mg/dL	34 mg/dL
	Glucose	50-80 mg/dL	53 mg/dL	62 mg/dL	119 mg/dL
	RBCs	0 cells/mm^3^	0 cells/mm^3^	0 cells/mm^3^	0 cells/mm^3^
	WBCs	0-8 cells/mm^3^	1 cells/mm^3^	4 cells/mm^3^	2 cells/mm^3^
	Neutrophils	0 cells/mm^3^	0 cells/mm^3^	3 cells/mm^3^	18 cells/mm^3^
	Lymphocytes	0-5 cells/mm^3^	33 cells/mm^3^	38 cells/mm^3^	73 cells/mm^3^
	Oligoclonal Bands	Absent	Absent	Absent	Absent
EEG Findings		Awake: desynchronized rhythms	Periodic lateralized discharges	Generalized periodic discharges	Diffuse slowing without clear periodic lateralized discharges
MRI Findings		Consistent signal intensities throughout the brain tissue without hyper- or hypo-intensities	Bilateral and symmetric cortical diffusion restriction (hyperintensity) in temporal, parietal, and occipital lobes (Cortical ribboning sign)	Bilateral and symmetric cortical diffusion restriction (hyperintensity) in frontal, temporal, and parietal lobes (Cortical ribboning sign)	Bilateral and symmetric diffusion restriction (hyperintensity) in all brain lobes (Cortical ribboning sign)
Lumbar Puncture		Neuron-specific enolase: <17 ng/mL; 14-3-3; Gamma (γ) protein: 0-1149 ng/mL; RT-QuiC: Positive*	14-3-3 γ: 6855 ng/mL (Elevated)	Neuron-specific enolase: 24.3 ng/mL (Elevated)	Neuron-specific enolase: 20.3 ng/mL (Elevated)

In this case series, we observed how classic symptoms of sCJD can initially mimic other neurologic conditions. MRI (cortical ribboning), CSF 14-3-3 protein and RT-QuiC are reliable early diagnostic tools. Hispanic populations may be underrepresented in CJD research due to diagnostic access barriers. Early diagnosis may improve the quality of care even though the prognosis remains unchanged. Larger epidemiological studies and genetic research in Hispanic cohorts are needed to clarify disease prevalence and potential predispositions.

## Conclusions

These cases represent an important contribution to the literature on prion diseases in underserved populations. While the clinical course of sCJD remains universally fatal, timely diagnosis enables more efficient care, minimizes unnecessary interventions, and supports families through a devastating prognosis. Recognition of early signs, especially in populations with limited neurologic care, remains essential for equitable medical practice.
